# Acute kidney injury in Ureteric Stones:Single centre short term analysis

**DOI:** 10.12669/pjms.334.13345

**Published:** 2017

**Authors:** Huma Mamun Mahmud, Syed Mamun Mahmud

**Affiliations:** 1 Dr. HumaMamun Mahmud, MCPS (Medicine), F.C.P.S (Nephrology). Department of Nephrology, Life Care Hospital Musaffah, Abudhabi, UAE. Visiting Faculty, South Asian Institute of Urology and Nephrology (SAIUN), Suite No. 603, 6^th^ Floor Al Khaleej Tower, Shaheede-Millat Road, Karachi, Pakistan; 2 Dr. Syed Mamun Mahmud, FCPS(Urology), FEBU. Department of Urology, Life Care Hospital Musaffah, Abudhabi, UAE. Visiting Faculty, South Asian Institute of Urology and Nephrology (SAIUN), Suite No. 603, 6^th^ Floor Al Khaleej Tower, Shaheede-Millat Road, Karachi, Pakistan

**Keywords:** Acute kidney injury, Recovery, Ureteric stones

## Abstract

**Objective:**

To analyze acute kidney injury (AKI) frequency, risk factors and outcome in ureteric stone patients.

**Methods:**

This is an observational retrospective study performed in a single tertiary care centre in Abu Dhabi from October 2014 till August 2015. Convenient sampling was done on 152 consecutive patients who underwent decompression surgery (Ureterorenoscopy LASER Lithotripsy and DJ Stenting) for ureteric stones. Serum Creatinine was used to calculate creatinine clearance by cock croft Gault formula in all patients before and after procedure. Analysis was done on SPSS version 17.

**Results:**

Out of 152 patients who under went decompression surgical procedure for ureteric stones AKI was seen in 49(32.2%). Patients with AKI were found to be more higher age, increasedweight, bilateral stones, lower ureteric stones and with co morbidities in comparison to those who were without acute kidney injury. Patients developing AKI, 89.7% recovered either partially(20.4 %) or completely (69.3%).

**Conclusion:**

AKI is seen more in ureteric stone patients with older age, increased weight, bilateral stones, lower ureteric stones and with co morbidities. Recovery is good when obstruction is relieved.

## INTRODUCTION

Urolithiasisisa well known entity for centuries. Archeologist have found treatment of urinary stone mentioned in old Egyptians medical writings.^1^-^3^ Over all probability of humans to develop a urinary stone is variable and is influenced by a number of factors including geographical area, race, socioeconomic status and dietary habits.^4^ Prevalence of urinary stone is increasing worldwide as reported byVictoriano R etal. in their review.^5^ It may be because of changing dietary pattern. Same paper also reveals that peak incidence was found from 40-49 years age group and men were affected more than females. In Iran and in America peak prevalence occurred between 60-69 years age group. Gender ratio varies from 2.5:1 in Japan to1.15:1 in Iran, stones were found highest among white man and lowest among Asian women.^5^

It is reported that 12% of men and 6% of women will have ureteric colic once in their lifetime. Majority of such colic do not require intervention. However 25% of patients will have recurrence of stones. The probability of spontaneous passage of stone is linked to its size and location. Stones ≤ 4mm (95-98%) and Middle & lower ureteric stones have higher probability of spontaneous passage.^6^

Stones are reported to be more common with consumption of starchy food derived from corn, increasing obesity, high fructose consumption.^7^,^8^ Less fluid and less calcium consumption are also linked with more stones^9^ so is more intake of sodium, oxalate and animal proteins all of which increases risk of having stones.^10^,^11^ Climate change of global warming also increasing stones.^12^

Prevalence of stone disease varies from 1-20%.^13^ Although kidney failure does not commonly results from nephrolithiasis, however it is responsible for a small number of causes of renal failure and the risk is more with stone in a solitary kidney and in patients with other risk factors including preexisting renal damage. In one study the incidence of AKI in ureteric stones is reported as 0.72%.^14^

In another study from Pakistan involving 2838 patients, 278(9.7%) developed acute renal failure and recovery after procedure was seen in 72% patients either complete or partial.^15^ Number of studies have been done to see incidence of renal failure and to see recovery of renal functions after removal of stones. Another study has compared that recovery of renal functions is complete (100%) and better in children less than two years old.^16^ In another study recovery in renal functions was found 94% in olderchildren of 3 yearsage.^17^

Removing stone does improves renal functions by removing obstruction and infection.^18^ Nephrolithiasis is associated with increased risk of CKD and ESRF shown in a number of studies as defined in a review article, in dependent of other risk factors for CKD.^19^

Our study is a simple observational study retrospectively done on all our patients who underwent stone removing procedures andwe observed renal function in them prior to and afterthe procedure to determine frequency of acute renal injury in ureteric stone patients and to see out come in our patients.

## METHODS

This study is an observational study done in a private tertiary care Centre in Abu Dhabi from Oct 2014 till August 2015. Study includes all patients who went forUreterorenoscopyLaser lithotripsy DJ stenting (URS LASER DJS) for ureteric stones during specified time period. Total number of patients assessed during this period was 152.

Sampling was done by convenient sampling in series on all those who went through decompression surgery for ureteric stones. Study was performed after getting approval from the Hospital Ethics Committee.

Patients were divided in to two groups, Group-I with AKI and Group-II without AKI. For each patient included in study demographic data was obtained including age & gender, history was noted for co morbidities like diabetes mellitus, hypertension, heart disease and weight was noted on first registration. Lab includes serum Creatinine (Cr) measurement before and after decompression procedure in all patients.

CreatinineClearance (Crcl) was calculated from Cockcroft and Gault formula (CCGF) on all patients using Serum Creatinine (Cr) values. Serum Cr was measured by using Jaffe method on Cobas 6000 machine. All included patients already were diagnosed ureteric stone on ultra sound and or CT abdomen without contrast. Lab tests of serum Cr was repeated post decompression procedure within one month on all patients and again Crcl was calculated from CCGF.

Acute kidney injury was defined by calculated Crcl of less than 90ml/min by CCGF at time of registration or an increase in serum Cr of more than 26micromol/l from baseline Cr if available. Recovery was defined as improvement in clearance complete when Crcl reaches normal value or partial when Crcl improves more than 20% but did not reaches normal post procedure.

All the data was retrieved from SAP Soft Ware used in Nephrology and Urology clinics in the same Institute. Data was analyzed with SPSS version 17, frequencies were applied for Categorical data and mean ±SD was used for defining numeric variables.

## RESULTS

Total number of patients included in study were152. Mean age was 35.84±8.745 with range of 21-64 years. All patients were males. All included patients were from three countries Pakistan, India and Bangladesh. Demographic data is mentioned in Table-I.

**Table-I:** Demographic data of study patients.

	*Total patients*	*Patients developing AKI*	*Patients with no AKI*
Number	152	49(32.2%)	103(67.7%)
Age	35.84±8.745	40.92±9.16	33.42±7.43
Gender	All males	All males	All males
Weight	72.09±10.15	89.67±9.99	73.24±10.07

(Percentage in parenthesis).

Demographic data shows mean weight was slightly higher in patients with AKI Vs non AKI (89.67±9.99kgVs 73.24±10.07 Kg); Mean age was also higher in AKI group Vs non AKI group(40.92+9.16 years Vs 33.42±7.43 Years). AKI was seen in 32.2% of patients. Patients developing AKI, 89.7% recovered either partially (20.4%) or completely (69.3%).

Among those with AKI most of them 42/49 were with Crcl from 60-89ml/min, 5/49 were with creatinine clearance between 30-59ml/minute and only two were with clearance below 30 ml/min. Both of patients with Crcl below 30 ml/minute were with co morbid conditions and recovery was partial in both patients.

Risk factors analysis is shown in Table-II, presence of co morbid conditions, lower ureteric stones and bilateral stones were seen more in patients with AKI. Data analysis is shown in Table-III.

**Table-II:** AKI Risk factors analysis in ureteric stone patients.

	*Total patient*	*patients with AKI*	*Patients with no AKI*
Number	152	49 (32.2)	103 (67.7)
Comorbidities (%)	20(13.2)	11(22.4)	9(8.7)
Complete Recovery (%)	NA	34(69.3)	NA
No recovery	NA	5/49(10.2)	NA
Partial recovery	NA	10/49(20.4)	NA
Upper ureteric stone	79(51.9)	19(38.7)	60(58.2)
Lower ureteric stone	73(48.1)	30(61.2)	43(41.7)
Rt ureteric stone	74(48.6)	23(46.9)	51(49.5)
Left ureteric stone	78(51.3)	26(53.1)	52(50.4)
Size of stone on ultrasound more than or equal to 1cm	58(38.1)	21(42.8)	37(35.9)
Bilateral stones	26(17.1)	12(24.4)	14(13.5)

(Percentage in parenthesis).

**Table-III:** Data analysis in ureteric stone patients.

*Name of variable*	*All patients*	*With AKI*	*Without AKI*
Mean Serum cr in mg/dl+SD before procedure	97.38±42.08	130.06±59.91	81.80±13.26
Cr cl by CCGF in ml/min before procedure	102.86±31.71	70.56±16.1	118.23±25.14
Mean Serum cr in mg/dl+SD after procedure	99.67±28.06	87.62±23.46	82.48±25.46
Cr cl by CCGF in ml/min after procedure	95.35±30.65	103.44±28.45	114.69±19.95

## DISCUSSION

Renal stones are common and increasing worldwide as shown in a number of studies.^5^ Prevalence of stone disease varies from 1-20%.^13^ Number of studies has been done earlier to analyze acute kidney injury in stone patients^5^,^7^,^15^ This study was done to observe the frequency of renal failure is ureteric stone patients and to see outcome of renal failure after the stone removing surgical procedure in our patients. It will be interesting to note the various pathogenesis contribute to AKI in these patients with ureteric stones including all classical Pre-renal, renal and post-renal etiology. As a first cause, it is noted that many patients in our study developed stone because of heavy sweating and poor water intake because logistics at work place. This may also happen with frequent vomiting with increasing severe episodes of colic. Likewise many continue taking analgesics mostly NSAID under prescription or un-prescribed because of feared loss of labor or again logistics at work place being remote or lack of transport availability. Last one refers to the disturbance in pressure dynamics in Bowman’s capsule, activating cascade of reaction elaborating substances injuring nephron.^20^ This is collectively called as Obstructive nephropathy. And this may happen with unilateral obstruction, as well as seen in our series and other reports. This is termed as Reflex Anuria. The pathogenesis in Unilateral and Bilateral Ureteral Obstruction requires a detailed note which is beyond the scope of this manuscript.

Our results are showing frequency of renal failure in 49/152(32.2%) patients. Renal failure is reported 9.7% in study done in Pakistan on 2838 out of which 278 were with renal failure.^15^ Another study shows frequency of renal failure 8% in stone disease.^21^ Among Indians reported frequency of chronic kidney disease is 17.2 %, with stone as a cause reported in 5.3%.^22^

High frequency of 32.2% reported by us might be because of our inclusion criteria we have only included those patients who presentswithsymptomatic ureteric stones while most of other studies include all stone patients. Over all stone disease incidence is high in gulf countries^23^, lot of factors affect stone disease here most important being hot weather. Labors are exposed to this extreme of temperature. Diet in this group of people who were studied is mostly outside food and often is mentioned to be unhealthy.

Bilateral renal stones are reported 5% of all urinary stones.^24^ In our research we found that among all patients’ bladderstones were found in 26/152(17.1%). We also found that 12/49 (24.4%) were having bilateral renal stones in AKI group and in non AKI group bilateral stones were seen in 14/103(13.5%). This is same as already reports mention that association of ARF is more with bilateral obstructing nephropathy^25^ however number of patients in our study is too small to draw definitive conclusions.

Our study has also shown that overall lower ureteric stones were more associated with AKI in (61%) than in non AKI patient (43%). Those who had renal failure 11/49 (22.4%) of them were with co morbidities like either diabetes mellitus hypertension or past history of renal stones.

Complete recovery was seen in 34(69.3%) of AKI patientswhere post procedure Crcl reaches normal of 90 and above while another 10 patients shows partial recovery where Crcl fails to reach normal valueof 90 and above however improves more than 20% from first Crcl, this make a total recovery seen in 44/49(89.7%). Hussain M etalfrom Pakistan reported recovery in 72%patients.^15^ Other studies have also reported recovery of 94-100% in AKI secondary to stone disease.^16^,^17^ High recovery rate in these studies as well as in ours, may be related to early intervention.

### Limitations of the study

It is a single center study with relatively less number of patients with only male participants as the study was done in a hospital attached to industrial area in Abu Dhabi which catches primarily labor community.

## CONCLUSION

Among ureteric stone patients AKI is seen in 32% of patients. Factors associated with AKI in our study were weight, age, presence of bilateral stones, lower ureteric stones and other co-morbidities. Majority of patients developing AKI, recovered either partially or completely after surgical intervention (URS LASER DJS) for decompression.

### Authors’ Contribution

**HMM:** Conceiving, study designing, data collection, manuscript writing, Review and final analysis.

**SMM:** Data collection, data interpretation, statistical analysis, discussion writing.

## References

[1] Tefkli A, Cezayirli F (2013). The history of Urinary stones: in parallel with civilization. Sci World J.

[2] Shah J, Whitfield HN (2002). Urolithiasis through the ages. BJU Int.

[3] Michell AR (1989). Urolithiasis-historical, comparative and pathophysiological aspects: a review. J R Soc Med.

[4] Lopez M, Hoppe B (2010). History, epidemiology and regional diversities of urolithiasis. Pediatr Nephrol.

[5] Victoriano R, Haluk A, Dean GA (2010). Kidney stones: A global Picture of prevalence, Incidence and associated risk factors. Rev Urol.

[6] Coll DM, Varanelli MJ, Smith RC (2002). Relationship of spontaneous passage of ureteral calculi to stone size an locations as revealed by unenhanced helical Ct. Am J Rhoentgenol.

[7] Taylor EN, Stampfer MJ, Curhan GC (2005). Obesity, weight gain, and the risk of kidney stones. JAMA.

[8] Taylor EN, Curhan GC (2008). Fructose consumption and the risk of kidney stones. Kidney Int.

[9] Hirvonen T, Pietinen P, Virtanen M, Albanes D, Virtamo J (1999). Nutrient intake and use of beverages and risk of kidney stones among ale smokers. Am J Epidemiol.

[10] Meschi T, Maggiore U, Fiaccadori E, Schianchi T, Bosi S, Adorni G (2004). The effect of fruits and vegetables on urinary stone risk factors. Kidney Int.

[11] Taylor EN, Curhan GC (2007). Oxalate intake and the risk for nephrolithiasis. J Am Soc Nephrol.

[12] Brikowski TH, Lotan Y, Pearle MS (2008). Climate related increase in prevalence furolithiasis in the United States. Proc Natl Acad Sci USA.

[13] Trinchieri A CG KS, Jun Wu K, Segura C.P, JW KS, Pak CY, Preminger GM, Tolley D (2003). Epidemiology. Stone Disease.

[14] Wang SJ, Mu XN, Zhang LY, Liu QY, Jin XB (2012). The incidence and clinical features of acute kidney injury secondary to ureteric calculi. Urol Res.

[15] Hussain M, Hashmi AH, Rizvi SA (2013). Problems and prospects of neglected renal calculi in Pakistan: can this tragedy be averted?. Urol J.

[16] Kotb S, Elsheemy MS, Morsi HA, Zakaria T, Salah M, Eissa MA (2013). Renal recoverability in infants with obstructive calcularanuria: Is it better than in older children?. J Pediatr Urol.

[17] Ziada AM, Sarhan OM, Habib EI, EITabie N, Sheemy M, Morsi HA (2011). Assessment of recoverability of kidney function in children with obstructive calcular anuria: multicentre study. J Pediatr Urol.

[18] Wood K, Keys T, Mufarriji P, Assimos DH (2011). Impact of stone removal on renal functions: A review. Rev Urol.

[19] Keddis MT, Andrew DR (2013). Nephrolithiasis and loss of kidney function. Curr Opin Nephrol Hypertens.

[20] Chevalier RL, Frobes MS, Thornhill BA (2009). Ureteral obstruction as a model of renal interstitial fibrosis and obstructive nephropathy. Kidney Int.

[21] Ullah K, Butt G, Masroor I, Kanwal K, Kifayat F (2015). Epidemiology of chronic kidney disease in a Pakistani population. Saudi J Kidney Dis Transpl.

[22] Singh AK, Farag YMK, Subramanian KK, Reddy SRK, Acharya VN, Almeida AF (2013). Epidemiology and risk factors of chronic kidney disease in India-results from SEEK(Screening and early Evaluation of Kidney Disease) study. BMC Nephrol.

[23] Wiliam GR (2012). Stone formation in Middle Eastern Gulf satates: A review. Arab J Urol.

[24] Schwartz BF, Stoller ML (2000). The vesical calculus. Urol Clin North Am.

[25] Alonso JV, Cachinero PL, Ubeda FR, Ruiz DJL, Blanco A (2014). Bilateral stones as a cause of acute renal failure in the emergency department. World J Emerg Med.

